# Is it tuberculosis mastitis or granulomatous mastitis? A thirteen-year experience at a university hospital

**DOI:** 10.55730/1300-0144.5637

**Published:** 2023-04-04

**Authors:** Handan İNÖNÜ KÖSEOĞLU, Mehmet Fatih DAŞIRAN, Reşit Doğan KÖSEOĞLU, Zekiye Ruken YÜKSEKKAYA ÇELİKYAY, Berati KALELİOĞLU

**Affiliations:** 1 Department of Pulmonary Diseases, Faculty of Medicine, Tokat Gaziosmanpaşa University, Tokat, Turkey; 2 Department of General Surgery, Faculty of Medicine, Tokat Gaziosmanpaşa University, Tokat, Turkey; 3 Department of Medical Pathology, Faculty of Medicine, Tokat Gaziosmanpaşa University, Tokat, Turkey; 4 Department of Radiology, Faculty of Medicine, Tokat Gaziosmanpaşa University, Tokat, Turkey; 5 Department of Medical Pathology, Tokat State Hospital, Tokat, Turkey

**Keywords:** Tuberculosis mastitis, granulomatous mastitis, tuberculosis, breast tuberculosis

## Abstract

**Background/aim:**

Granulomatous mastitis (GM) is a rare inflammatory disease of the breast. Tuberculosis mastitis (TM), one of the causes of GM, is a rare form of extrapulmonary tuberculosis. The clinical, radiological, and histopathological findings of TM and GM are similar, and sometimes it is difficult to make a distinction between these disease states. In this study, we aimed to evaluate the clinical and radiological features, diagnostic techniques, treatment modalities and treatment outcomes of the patients with GM and TM.

**Materials and methods:**

The data of the patients with confirmed GM by histopathologic examination of biopsy specimens between 2007 and 2020 were retrospectively analyzed. Demographic features, main complaints, physical findings, radiological and laboratory data, treatment modalities, and treatment outcomes were recorded.

**Results:**

Sixty-eight GM patients with a mean age of 35.8 (18–63) years were evaluated. The patients had a mass lesion, pain, ulceration, and abscess in their breasts. All of the cases were female. Ultrasonographic examinations were performed on 62 cases. Abscess and/or sinus tract formation was detected in 34, heterogeneous hypoechoic mass in 15, heterogeneous parenchyma or parenchymal edema in 15, axillary lymphadenopathy in 18 and cysts in 13 patients. A total of 10 patients were lost to follow-up. Twenty-six patients underwent surgery for their breast lesions or had antibiotherapy (n = 13) or corticosteroid therapy (n = 7). Eleven (16.1%) patients were diagnosed with TM. These patients were evaluated by clinical examination, chest radiography, and tuberculin skin test. Acid-fast bacilli (AFB) staining and culture were negative in all cases. The diagnosis of TM was based on histopathological evaluation results. Eight of the 11 patients achieved complete remission with antituberculosis treatment.

**Conclusion:**

The etiological diagnosis of GM must be based on a multidisciplinary approach. Tuberculosis mastitis should become a part of differential diagnosis of breast diseases in populations with high incidence of tuberculosis.

## 1. Introduction

Granulomatous mastitis (GM) is a rare inflammatory condition of the breast. It has two forms: idiopathic GM and secondary GM. Idiopathic GM is defined as GM without any other causes, and secondary GM can develop due to tuberculosis, sarcoidosis, Wegener’s granulomatosis, foreign body reaction, vasculitis, and fungal infections [1]. Tuberculous mastitis (TM) is a rare type of extrapulmonary tuberculosis and is often clinically misdiagnosed as other breast lesions. It may occur as a part of systemic tuberculosis or as an isolated lesion [2]. Both TM and GM usually occur in women of reproductive age and may be associated with lactation or the postpartum period. Clinically, GM presents as breast masses, inflammation, local pain, tenderness, abscesses, and fistulae [1]. These clinical signs are also seen in TM. Many patients have a mass fixed to the skin or underlying structures and retraction of the nipple. In those situations, GM might be easily confused with cancer. Therefore, early and correct diagnosis is very important for appropriate treatment. In this study, we aimed to evaluate the clinical and radiological features, diagnostic techniques, treatment modalities, and treatment outcomes of the patients with GM and TM, in our hospital.

## 2. Materials and methods

### 2.1. Study design and setting

The patients with confirmed GM by histopathologic examination between January 2007 and June 2020 at Tokat Gaziosmanpaşa University Faculty of Medicine Department of Pathology were retrospectively analyzed. We noted demographic features, age, sex, medical family history, main complaints, tuberculosis (TB) contact history, systemic symptoms, physical findings, marital status, gestation, lactation, tuberculin skin test (TST) results, diagnostic interventions, chest Xray scans, the existence of a TB type other than breast TB, medical and surgical treatments, duration of antiTB treatment, and treatment outcomes. The chest X-ray, ultrasonography (US), mammography (MG), and magnetic resonance imaging (MRI) findings were obtained from patients’ files. Ziehl-Neelsen staining and Lowenstein-Jensen culture were performed on smears for a microbiological diagnosis of tuberculosis. In addition, Gram staining and culture of the aspiration samples were done.

The Ethics Committee of Gaziosmanpaşa University Faculty of Medicine, Tokat, Turkey approved the study. The document number is 20.KAEK.233.

### 2.2. Statistical evaluation

Descriptive analyses were made to give information about the general characteristics of the study groups. Quantitative variables were presented as arithmetic mean ± standard deviation and qualitative variables as numbers and percentages. Analyses were performed using statistical software SPSS 22.0 (Chicago, IL, USA).

## 3. Results

A total of 68 patients diagnosed with GM were identified during the period between 2007 and 2020. All of the patients were female. The mean age of the patients was 35.82 ± 9.13 years (age range, 18–63 years). The patients had a breast mass, tenderness, pain, swelling, ulceration, and abscess in one or two breasts. Unilateral breast involvement was observed in 62 of 68 patients, including involvement of the right breast in 32 (47.1%), and the left breast in 30 (44.1%) patients. In addition, 6 (8.8%) patients presented bilateral breast involvement.

**Figure 1a:**
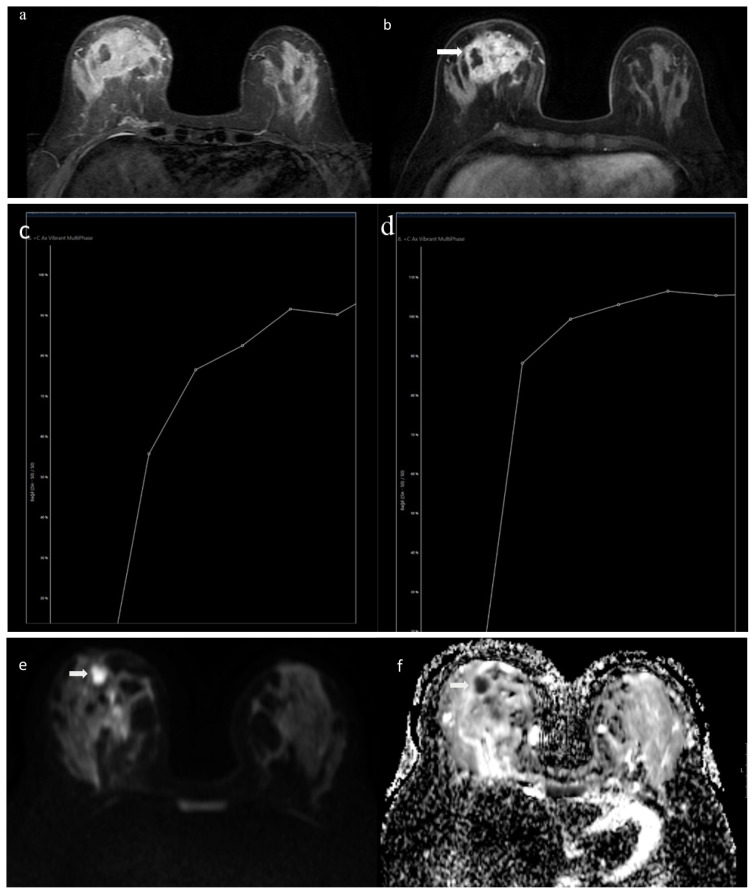
A 37-year-old female mother with right breast lump and bilateral breast pain. Axial STIR-weighted MR image shows a 5.5 × 4 cm (APxKK), hyperintense, mass-like lesion at right breast. **1b**. Axial fat supressed T1-weighted MR image shows regional heterogeneous contrast enhancement including abscess formation (white arrow). **1c**. Time intensity curve demonstrate progressive increase (Type I) contrast pattern. **1 d**. Time intensity curve demonstrate plateau (Type II) contrast pattern. **1e–1f**. Diffusion weighted MR images show the restriction of diffusion (white arrows).

US was performed on 62 patients, and some of the patients had undergone MG and MRI. An abscess and/or sinus tract formation was observed in 34 (54.8%) patients. A heterogeneous hypoechoic mass (or confluent mass) with indistinct, lobulated, or angular margins was identified in 15 (24.1%) patients. We observed heterogeneous parenchyma or parenchymal edema in 15 (24.1%), and axillary lymphadenopathy in 18 (29%) women. Thirteen patients (20.9%) had cysts including complicated cysts in 7 (53.8%) and complex cysts in 4 (30.7%) cases. A representative example of an MRI examination performed for the diagnosis of GM is shown in Figures 1a–1f.

A total of 10 patients were lost to follow-up. Twenty-six patients underwent surgical interventions (excision/drainage/segmentectomy) of the breast lesions, while others received anti-TB treatment (n = 11), antibiotherapy (n = 13), and corticosteroid therapy (n = 7). Treatment of one patient is still ongoing.

Aspiration/biopsy samples were examined for tuberculosis in only 29 (42.6%) of 68 cases. All of the samples were negative for acid-fast bacilli (AFB) on staining, as well as negative on culture (Lowenstein-Jensen culture media). Tuberculosis PCR analysis was performed in only one case which yielded negative results. Also, Gram staining was done in 34 patients, and any pathogenic microorganism could not be identified. Bacterial cultures were positive in only 4 cases.

Twenty-five of 68 patients with GM had consulted to the Department of Pulmonary Diseases. All of the patients were evaluated with clinical examination, chest X-ray, BCG vaccine scar, TST. Eleven (16.1%) patients were diagnosed as TM.

All TM patients had a BCG scar and TST yielded a mean skin induration of 13.18 ± 5.49 mm. Chest Xray scans were normal in all cases. No patient had pulmonary or extrapulmonary tuberculosis. Clinically, these patients had tender, erythematous, firm breast masses with or without sinus formation. Unilateral breast involvement was observed in 10 of 11 cases, including involvement of the right breast in 6 and left breast in 4 cases. Besides, one case presented with bilateral breast involvement.

All TM patients had undergone ultrasonographic examination which showed hypoechoic collections, solitary masses, fistula tracts, inflammatory changes, dilated ducts, and increased skin thickness. Five patients had an associated axillary lymphadenopathy.

**Figure 2:**
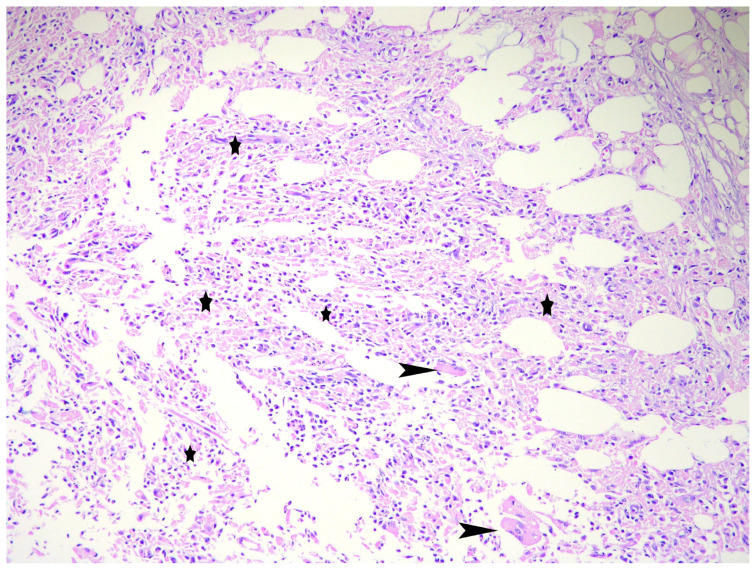
Histopathologic findings in a tuberculous mastitis case. A caseating granuloma consisting of epithelioid histiocytes (asterisks), and multinucleated giant cells (arrowheads) (hematoxylin & eosin, Χ20).

Staining for acid-fast bacilli and microbiologial cultures were not remarkable and yielded negative results in all cases. Upon histopathological examination of breast tissue, granulomatous inflammation with typical caseous necrosis was observed in 1 (Figure 2), noncaseous granulomatous inflammation in 9, and mastitis and fat necrosis inflammation in 1 case. Granulomas consisted of epithelioid histiocytes, Langhans giant cells, lymphocytes, and plasma cells.

Tuberculostatic treatment was administered according to the Turkish national guidelines [3]. The TB treatment consisted of 2 months of isoniazid (H) + rifampin (R) + ethambutol (E) + pyrazinamide (Z) (HRZE regimen), followed by 4 months of isoniazid (H) + rifampin (R) (HR regimen). (H: 300 mg; R: 600 mg; Z: 2000 mg; E: 1500 mg). As a result of anti-TB treatment of 11 patients, 8 patients achieved complete remission, 1 patient had incomplete remission and retained a mass in her breast, while 1 patient reapplied with an abscess in the contralateral breast 6 years later, and the remaining patient was lost to follow-up without any information about the result of her treatment.

## 4. Discussion

Granulomatous mastitis representing approximately 0.025%–3% of all surgically treated breast diseases is an uncommon breast lesion that was first described in 1972 [2, 4]. Various etiologic factors cause GM including infections such as tuberculosis, leprosy, diphtheria, cat-scratch disease, syphilis, blastomycosis, histoplasmosis, cryptococcosis, schistosomiasis, actinomycosis, filarial infection, autoimmune diseases including Crohn’s disease, sarcoidosis, Wegener’s granulomatosis, giant cell arteritis, plasma cell mastitis as well as a reaction to foreign bodies [2, 5]. Granulomatous mastitis usually affects women with a history of breastfeeding or recent birth. Hormonal alterations during these processes, milk secretion, and inflammation have an effect on disease pathophysiology [6]. The most common clinical presentation is a solid, unilateral breast mass, often associated with inflammation of the skin. The disease course is characterized by slow resolution, occasionally with intermittent episodes of abscess or sinus formation [7]. The clinical presentation of the mass lesion appears as a rigid breast lump with nipple retraction in some patients, mimicking a case of carcinoma [8–10]. Regional lymphadenopathy may be present in up to 15% of cases [11]. In our study, the most common presenting symptom was a solid mass, which was detected in all of the patients. Some of the patients had ulcerations, abscesses, fistula formations, while axillary lymphadenopathy was present in 24 (38.7%) cases.

There is no clear clinical consensus regarding the ideal therapeutic management of GM. Percutaneous or surgical drainage of abscesses, extensive surgical excision of the lesion, medical treatment with antibiotics, corticosteroids, immunosuppressive drugs are commonly applied in the management of GM. The recurrence rate is high even after mastectomy. Administration of corticosteroids for large lesions before surgery is recommended to help minimize lesion size; also they are used as a primary postsurgical treatment to prevent GM recurrences [6]. Topical corticosteroids were also reported to be effective in the treatment of GM [12]. Observational management may also be an acceptable approach because more than half of the patients with GM who did not receive any treatment were found to achieve complete remission [13, 14]. Close follow-up should be preferred particularly in patients with a mild disease without signs of inflammation. Bouton et al. considered GM as a self-limiting benign condition, and they found the average time to resolution to be 7.4 months [15]. In the current study, 26 patients underwent surgical interventions (excision/drainage/segmentectomy) for the breast lesion. Surgery was performed in cases with abscess, nonhealing sinus, and fistula formation at first admission or follow-up. Thirteen patients had antibiotic, and 7 had corticosteroid therapy.

Fine needle aspiration cytology (FNAC) is the primary diagnostic test for breast lesions. However, FNAC may not always differentiate between idiopathic GM and other granulomatous diseases of the breast, and a specific diagnosis may require histopathological examination of the samples, microbiological investigations, and their correlation with clinical findings. Idiopathic GM is actually a diagnosis of exclusion, so all causes of granulomatous inflammation must be actively excluded by performing special tests. Therefore, the etiological diagnosis of GM must be based on a multidisciplinary approach. Distinguishing idiopathic GM from TM is highly important. Treating tuberculosis with steroids would aggravate the infection, whereas giving unnecessary antituberculosis drugs in patients of idiopathic GM may cause numerous side effects [16].

Tuberculosis mastitis is a rare type of extrapulmonary tuberculosis. In one study, the rate of TM was 1.25% among all tuberculosis cases, and 2.27% of all extrapulmonary tuberculosis cases [17]. The rate of TM has been reported as 13.3%, 14.7%, 23.8%, 40% of GM cases, in various studies [18, 19, 20, 2]. In our study, TM was seen in 16.1% of the cases. Tuberculosis mastitis commonly affects female patients of reproductive age (range, 20–40 years). In pregnant and lactating women, the increased vascularity of the breast with dilated ducts predispose to infection. There were no pregnant or lactating women in the patient cohort of the present study, and the mean age of the patients was 36.4 years. Tuberculosis mastitis can be seen as a component of a systemic disease or per se. In a review, tuberculosis mastitis was revealed in 18.7% of the cases with a history of TB [21]. In the current study, none of the patients had a history of TB or systemic involvement.

Tuberculosis involvement of breast commonly occurs by dissemination of the bacilli via lymphatic or hematogenous routes. The lymphatic route is the most likely route of breast involvement which occurs by retrograde extension from the axillary lymph nodes. This hypothesis is supported by the involvement of axillary nodes, frequently ipsilateral nodes, in 50% to 75% of TM cases. In the present study, axillary lymphadenopathy was observed in 45.4% of cases. Hematogenous spread is rare and occurs in cases of disseminated tuberculosis. Contiguous spread may occur from the ribs or pleural space [16]. In our study, the only organ involved was the breast.

Tuberculosis mastitis is divided into groups of the nodular, disseminated and, sclerosing type. The nodular type is the most common type and usually presents as solitary or multiple well-circumscribed, slowly growing painless masses. In addition, the disseminated type is seen due to spread of highly virulent organisms or in immune-deficient patients. This form usually manifests as multiple foci of infection, which may later lead to abscess and sinus formation. The breast may be tender and painful. Finally, in the sclerosing type, extensive fibrosis is a dominant feature, which may lead to secondary retraction and atrophy of the breast. This form is usually seen in older patients. The asymmetry between the two breasts and reduction in breast volume may occur in these patients [22].

The commonly reported presentation of TM is a breast lump and breast abscess. Other findings are sinus or fistula, skin ulceration, and nipple retraction. The most common constitutional symptoms are pain and fever. Clinical findings such as constitutional symptoms, mobile breast lumps, multiple sinuses and, retracted nipple, especially in young multiparous or lactating women, are predictive but not specific features of TM [23]. In our study, the commonly observed symptoms were mass, pain, swelling and abscess formation in the breast. Nipple retraction or discharge was not observed in any of the included patients. Involvement of the right breast was observed in 6 of 11 cases, which is consistent with previous reports [21].

Ultrasonography is a valuable diagnostic modality in suspect cases of TM, both for evaluating the extent of the disease and also for performing an image-guided biopsy and percutaneous drainage of an abscess. In advanced stages of breast TB, ultrasonography is usually more helpful than mammography in distinguishing carcinoma from TM. On US, the presence of multiple intercommunicating breast abscesses, sinus tract formation(s), and multifocal breast lesions favor the diagnosis of TM rather than a malignant lesion [24].

In the current study, US revealed the presence of abscess, fistula tracts, heterogeneous hypoechoic mass, heterogeneous parenchyma or parenchymal edema, axillary lymphadenopathy, and cysts. Five patients had associated axillary lymphadenopathy. It is important to be aware of the fact that breast carcinoma and TM may occasionally coexist. Tulasi et al. have reported a case of infiltrating ductal carcinoma of the breast and metastasis to the axillary lymph node with evidence of tubercular granuloma in the same lymph node [25]. Farrokh et al. reported coexistence of breast carcinoma and TM in one breast [26]. Similar case reports have been published in the literature [27, 28]. It is important to remember that the recognition of TB does not exclude the presence of concomitant breast carcinoma [29].

Clinical and radiological features are nonspecific for the diagnosis of TM. Fine needle aspiration cytology, tru-cut biopsy, open biopsy, and histopathological examination are important diagnostic tools. Fine needle aspiration cytology is the most widely used initial invasive diagnostic method. The gold standard for the diagnosis of TM is the detection of the etiologic agent using Ziehl-Neelsen staining or culturing; however, the AFB test rarely yields positive results. The detection rates of microbiological tests for AFB, and isolation in culture have been reported as 26.6%, and 25.9%, respectively. For this reason, in tuberculosis-endemic countries, the finding of granuloma in fine-needle aspiration biopsy material warrants empirical treatment for tuberculosis even in the absence of positive AFB and culture results [16]. Khanna et al. reported 52 cases of TM within 15 years. The diagnosis of TM had been confirmed using FNAC or histological examination in all patients [30]. Another study by Al Marri et al. reported 13 multiparous women with TM within a 10-year period, who were diagnosed based on histological examination of biopsy specimens [31]. In a systematic review, diagnosis of TM was based on histopathology results and confirmed response to anti-TB therapy. The authors claimed that histopathological examination appears to be a more practical use than bacteriological tests [21]. The Tuberculosis Diagnosis and Treatment Guide published by the Ministry of Health in our country states that the most important finding for the presence of the tuberculosis is the “granulomatous reaction”. The presence of “caseous necrosis” is not absolutely necessary [32]. In our study, the diagnosis of TM was made on the basis of histopathological findings and TST positivity. Although typical caseous necrosis was observed in only one case, granulomatous inflammation was present in all cases. Lesions in the breast completely resolved in 8 of 11 cases with anti-TB treatment, and the diagnosis of TM was confirmed with the response to treatment.

Bacterial culture is the gold standard for the diagnosis of active tuberculosis. However, it is time-consuming and requires a high-quality sample. In recent years, several molecular diagnostic methods have been developed. Polymerase chain reaction (PCR) method is generally used to detect tuberculosis in culture- and microscopy-negative samples or to differentiate and typing *M*. *tuberculosis* from atypical mycobacteria, and to detect genetic mutations that cause resistance to antituberculosis drugs. This method enables the detection of *M. tuberculosis* without culturing by amplifying the DNA or RNA segment of the bacteria. PCR method can be studied in blood, sputum, gastric aspiration fluid, urine, cerebrospinal fluid, pleural fluid, and other body fluids and tissue samples. It provides early diagnosis and treatment opportunity [33]. Real-time PCR is a technique in which amplification is measured simultaneously with a fluorescent signal and allows quantitation. In our study, PCR analysis was performed in only one case which yielded a negative result.

Fluorescent in situ hybridization (FISH) method is a fast, practical, and inexpensive method that allows identification of different species of mycobacteria, especially the tuberculosis complex, and their associates, without the need for isolation and amplification of the target gene [34].

The Xpert MTB/RIF is a cartridge-based, automated diagnostic test that can identify *M*. *tuberculosis* DNA and resistance to rifampicin (RIF) by nucleic acid amplification techniques (NAAT) in less than 2 h. Results from field demonstration studies have demonstrated that a single Xpert MTB/RIF test can detect *M*. *tuberculosis* in 99% of the patients with smear-positive pulmonary tuberculosis and more than 80% of the patients with smear-negative pulmonary tuberculosis [35].

Previously, mastectomy was the common treatment of TM; however, in recent years, since anti-TB drug therapy is highly successful, surgical intervention is rarely performed [36]. The success rate of medical therapy approaches 95% in most series with 6 months of anti-TB therapy. Some authors prefer the 9-month regimen due to a lower relapse rate in general [16]. Liu et al. evaluated 22 patients with GM where anti-TB treatment was performed in 19 patients, after excluding malignancies, sarcoidosis and, Wegener’s granulomatosis. Among them, 18 patients achieved complete remission, and no recurrences or new lesions were observed in the long-term follow-up in all patients [6]. Another study by Khurram et al. reported that all 22 patients with TM were cured with anti-TB treatment [37]. Pregnancy and lactation do not change tuberculosis treatment regimen [3]. Available data do not suggest any significant adverse maternal and fetal effects or need for dose adjustment in pregnancy. Pregnant women on isoniazid should take pyridoxine to prevent peripheral neuropathy. Mothers can breastfeed their babies from an uninfected breast [38]. Infection with multidrug-resistant tuberculosis (MDR) has been reported. Therapy with a combination of first- and second-line drugs that include kanamycin, ofloxacin, ethionamide, para-aminosalicylic acid (PAS), pyrazinamide, and isoniazid should be instituted [39, 40].

Surgical intervention may be required in case of unresponsiveness to medical treatment, and for painful ulcerated and also residual lesions. In our study, 8 of 11 patients achieved complete remission with anti-TBC chemotherapy. In one patient, a mass lesion was retained in the breast, one patient reapplied with an abscess in the contralateral breast 6 years later, and one patient was lost to follow-up, so no information was obtained about her treatment outcomes.

The main limitation of this study is that the patients’ records were retrospectively analyzed. Therefore, some data were not available. The results of the patients who were lost to follow-up could not be obtained. Microbiological data were very limited in the file records, so AFB and culture positivity rates could not be evaluated while making the diagnosis of tuberculosis in our patients.

In conclusion, all GM cases are not related to tuberculosis. Tuberculosis mastitis is an uncommon disease even in countries with high incidence of tuberculosis. The etiological diagnosis of GM must be made using a multidisciplinary approach. Radiological, cytopathologcal, and microbiological methods of diagnosis should be used in combination. Clinical signs related to tuberculosis should be carefully investigated, especially in endemic regions.
